# Luminescent Properties of Lanthanoid-Poly(Sodium Acrylate) Composites: Insights on the Interaction Mechanism

**DOI:** 10.3390/polym12061314

**Published:** 2020-06-09

**Authors:** Alan F. Y. Matsushita, María José Tapia, Alberto A. C. C. Pais, Artur J. M. Valente

**Affiliations:** 1 CQC, Department of Chemistry, University of Coimbra, 3004-535 Coimbra, Portugal; alanmatsushita@hotmail.com (A.F.Y.M.); pais@ci.uc.pt (A.A.C.C.P.); 2 Department of Chemistry, Universidad de Burgos, 09001 Burgos, Spain; mjtapia@ubu.es

**Keywords:** fluorescence quenching, polymer composite, lanthanoid complex, metal-organic gel, responsive gel

## Abstract

The interaction between polyelectrolytes and metal ions is governed by different types of interactions, leading to the formation of different phases, from liquid state to weak gels, through an appropriate choice of metal ion/polyelectrolyte molar ratio. We have found that lanthanide ions, europium(III) and terbium(III), are able to form polymer composites with poly(sodium acrylate). That interaction enhances the luminescent properties of europium(III) and terbium(III), showing that Eu^3+^/poly(sodium acrylate) (PSA) and Tb^3+^/PSA composites have a highly intense red and green emission, respectively. The effect of cations with different valences on the luminescent properties of the polymer composites is analyzed. The presence of metal ions tends to quench the composite emission intensity and the quenching process depends on the cation, with copper(II) being by far the most efficient quencher. The interaction mechanism between lanthanoid ions and PSA is also discussed. The composites and their interactions with a wide range of cations and anions are fully characterized through stationary and non-stationary fluorescence, high resolution scanning electronic microscopy and X-ray diffraction.

## 1. Introduction

Metal-organic materials have received great attention in recent years, because of the metal-ligand interaction, which can form three-dimensional structures, such as metal-organic frameworks (MOF) and metal-organic gels (MOG); these materials have already presented promising applications for supercapacitors, sensing, catalysis and optoelectronics [1,2,3,4,5]. MOG preparation is simpler, involving hydrogen bonding interactions, π-π stacking, van der Waals forces and coordination bonds under mild conditions to form self-assembled supramolecular structures [6,7]. On the other hand, MOF usually display a highly crystalline structure, therefore requiring a more time-consuming preparation [8]. MOG, on the contrary, are characterized by extended structures, essentially driven by metal-ligand interactions, where the polymer (ligand) acts as a gelling agent [9]. These materials may show selective sensing to different stimuli including cations and anions [6,10]. Some advantages of these MOGs include high sensitivity, availability, fast response to a stimulus, low cost (small amounts of probe is required) and fluorescence, which is a technique frequently used in the sensing field [11]. In this regard, lanthanoid complexes have interesting spectroscopic properties—such as large Stokes shift, narrow emission bands, long emission lifetimes and high emission quantum yields [12]—being a good alternative as sensors for detection of cations, anions and biomolecules. In fact, the detection of metals, such as copper(II)—a relevant ion for biochemical and environmental issues [13,14,15]—can be done by different methods, including electrochemical methods, UV-vis spectroscopy, atomic absorption, inductively coupled plasma and atomic emission spectrometry (ICP-AES) [16,17,18,19]. These methods generally require costly equipment and the specific preparation of samples. Hence, the development of fluorescent sensors for metal ions has increased in recent years [20,21,22,23].

Following our previous work [24], we describe here the synthesis and characterization of two new polymer composites consisting of lanthanoid metal ions (europium and terbium) and a polyelectrolyte. The luminescent properties of these compounds were assessed, making it possible to have an insight into the mechanism of interactions between lanthanoid ion and the polyelectrolyte. Additionally, the effect of cations with different stable valences and good solubility were assessed to better understand the hypothetical competition mechanism with lanthanoid ions on the poly (sodium acrylate)-Ln^3+^ interaction; moreover, and once that salts with different monovalent anions were used, a series of monovalent anions were also studied. That made it possible to shed light on the possible application of these matrices for sensing, remediation or anti-counterfeiting. 

## 2. Materials and Methods

### 2.1. Reagents

All chemicals were commercially available and used without further purification. The following salts: europium(III) chloride hexahydrate, terbium(III) chloride hexahydrate, aluminum hydroxide hydrate, calcium chloride dihydrate, cerium(III) chloride heptahydrate, chromium(III) chloride hexahydrate, copper(II) chloride dihydrate, mercury(II) thiocyanate, potassium chloride, sodium chloride, sodium acetate, sodium bromide, sodium cyanide, sodium fluoride, nickel nitrate hexahydrate, lead(II) nitrate, zinc chloride and poly(sodium acrylate) (PSA, $\bar{M_{w}}$ = 2100 g mol^−1^) were purchased from Sigma-Aldrich (Steinheim, Germany). Sodium acetate, magnesium nitrate hexahydrate, were obtained from Fluka (Gillingham, UK) and anhydrous aluminum chloride, sodium nitrite and potassium thiocyanate were purchased from Merck (Darmstadt, Germany). Milli-Q water was used in all the experiments. 

### 2.2. Eu^3+^/PSA and Tb^3+^/PSA Composite Solutions

The Eu^3+^/PSA and Tb^3+^/PSA composites were prepared by dissolving PSA (0.26 mol dm^−3^, in terms of polymer repetition units, whose repetitive unit molecular weight is 108.08 g mol^−1^) in milli-Q water, and the subsequent dropwise addition of europium or terbium chloride hexahydrates (0.026 mol dm^−3^), under continuous magnetic stirring. The same method was used elsewhere [24]. The pH of the solutions was adjusted at around 7.5, by adding either NaOH or HCl (0.1 mol dm^−3^), due the p*K*_a_ value of PSA and the solubility of Eu^3+^ and Tb^3+^ [25]. It has been found that the addition of salts did not change the solution pH. Additionally, the stability of solutions was verified spectrophotometrically for at least 2 weeks after preparation [24]. 

### 2.3. Preparation of Metal-Organic Gels

Freshly prepared solutions of Eu^3+^/PSA and Tb^3+^/PSA were used for the luminescent studies. To investigate the selectivity of the composites towards different metal ions, aliquots of metallic ion stock solutions (Al^3+^, Ca^2+^, Ce^3+^, Cr^3+^, Cu^2+^, Hg^2+^, K^+^, Mg^2+^, Na^+^, Ni^2+^, Pb^2+^ and Zn^2+^) were added to Eu^3+^/PSA and/or Tb^3+^/PSA solution. The final concentration of metal ions in the solution was fixed at 3.33 mM. The solution was mixed using an ultrasonic bath for 30 min, before the emission spectra was recorded. All the spectra were registered at 25 °C.

### 2.4. Apparatus and Characterization Methods of Eu(III)/PSA and Tb(III)/PSA

The UV–vis spectra of solutions were recorded on a Shimadzu 2450 UV–vis spectrophotometer (Kyoto, Japan). 

Emission spectra were recorded with a Fluoromax-4 spectrofluorometer (Kyoto, Japan), in a right-angle configuration, with excitation at 395 nm (Eu^3+^) and 273 nm (for Tb^3+^), and emission spectra scanned between 550 and 750 nm and 450 and 650 nm, respectively. Excitation and emission slits of 0.5 and 1.0 nm, respectively, were used.

Tb^3+^ and Eu^3+^ luminescence lifetimes were measured with the multi-channel scaling mode (MCS) counter module (TCC2) of the FLS980 spectrometer (Edinburgh Instruments, Livingston, UK), using a microsecond flash Xe-lamp (μF2) as light source. The decays were registered by exciting the composites at 395 nm and 270 nm and recording the emission at 613 nm and 543 nm for Eu^3+^/PSA and Tb^3+^/PSA, respectively. The decays were analyzed with the software of the equipment.

High Resolution Scanning Electronic Microscopy (HR-SEM) micrographs were obtained using a ZEISS Merlin scanning electron microscope (Oerzen, Germany), operating under low vacuum at 2kV. Elemental analysis on microscopic sections of composites was performed by Energy Dispersive Spectroscopy—Oxford Instruments, Oxon, UK. The samples used in these techniques were previously frozen at −20 °C and then lyophilized (Free Zone 4.5-Labconco), before being sputter-coated with a thin gold layer.

X-ray diffractograms were obtained with freeze-dried samples with the (X-ray Diffractometer Rigaku Ultima IV, Tokyo, Japan) with the following characteristics: Cu-kα radiation (1.54 A) in the range of 5° to 120° at a scan speed of 2*θ*/min.

## 3. Results

### 3.1. Lanthanoid-PSA Interactions

**Figure 1:**
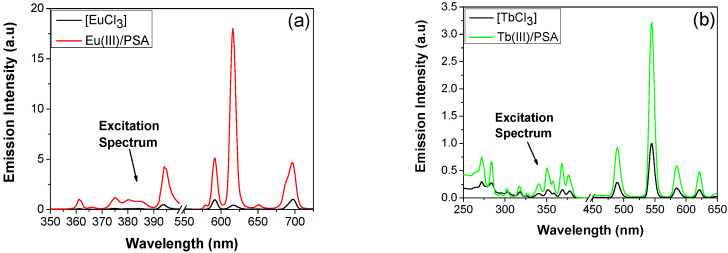
Excitation spectra (left side) and emission spectra (right side) of (**a**) EuCl_3_ and Eu^3+^/PSA (λ_em_ = 616 nm and λ_ex_ = 395 nm) and (**b**) TbCl_3_ and Tb^3+^/PSA (λ_em_ = 545 nm and λ_ex_ = 273 nm) in aqueous solutions at pH 7.2.

The Eu^3+^/PSA and Tb^3+^/PSA complexes, synthesized using a previous method [24], show an enhanced luminescence intensity in comparison to the free lanthanoides (see Figure 1). The metal-polymer interactions are likely due to the PSA carboxylic groups acting as chelators to the lanthanoid ions. 

From analysis of Figure 1, it can be observed that upon excitation at 395 nm Eu^3+^/PSA exhibits strong emission peaks at 580, 593, 616, 652 and 694 nm. These peaks can be assigned to the characteristic ^5^*D*_0_ → ^7^*F*_J_ (*J* = 0–4) transitions of the Eu^3+^ ion. The ^5^*D*_0_ → ^7^*F*_1_ transition is a magnetic dipole transition, which corresponds to the weak emission band at 593 nm, less sensitive to the coordination environment. Therefore, when Eu^3+^ ions occupy the non-inversion centers sites, the electric dipole transition ^5^*D*_0_ → ^7^*F*_2_ is dominant promoting red-light emission. By comparing the transitions ^5^*D*_0_ → ^7^*F*_2_ and ^5^*D*_0_ → ^7^*F*_1_, we obtain an intensity ratio *I*(^5^*D*_0_ → ^7^*F*_2_)/*I*(^5^*D*_0_ → ^7^*F*_1_) of about 3.4, thus indicating that Eu^3+^ occupies non-inversion centers [26,27]. Similarly, upon excitation at 273 nm, Tb^3+^/PSA displays four clearly resolved peaks at 490, 545, 585 and 623 nm, which can be ascribed to ^5^*D*_4_ → ^7^*F*_J_ (*J* = 6–3) transitions of Tb^3+^ ions. ^5^*D*_4_ → ^7^*F*_5_ is an electric induced dipole transition, which is characterized by the intense emission band at 545 nm, more sensitive to the coordination environment [28,29]. Additionally, the lanthanoid(III)-polymer interaction should lead to a concomitant dehydration of metal ions [30]. To have an assessment on such hypothesis, non-stationary fluorescence experiments were carried out, to allow the computation of the number of water molecules coordinating the lanthanoid; this can be done by comparing the fluorescence lifetime of Tb^3+^ and Eu^3+^ in aqueous and D_2_O solutions [30,31,32]. The decays of Tb^3+^ and Eu^3+^ (2.7 × 10^−2^ mol dm^−3^) luminescence were registered in the absence and in the presence of PSA (0.23 mol dm^−3^, in terms of monomer), both in H_2_O and D_2_O solutions. Good monoexponencial decays were observed in all the cases, from which lifetimes (*τ*) and decay constants (*k*, reciprocal lifetime) were obtained (Table 1). From this, the number of bound water molecules (*n*) was computed. It has been found that Eu^3+^ and Tb^3+^, in aqueous solution, are coordinated by nine water molecules, which is in good agreement with the literature data [31]. However, in the presence of PSA, the number of bound water molecules decrease to approximately three. Such a decrease in the number of water coordinated molecules have also been observed for lanthanoids interacting with poly(vinyl sulfate) [33] or sodium dodecyl sulfate [34]. It can be concluded that PSA can replace up to six lanthanoid water coordinated molecules, indicating a strong interaction with Ln^3+^ ions, partially inhibiting the efficient radiationless lanthanoid emission deexcitation pathway via energy transfer to OH vibrational overtones [31,35]. This fact explains the significant increase of Eu^3+^ and Tb^3+^ emission intensity upon complexation with PSA (Figure 1).

**Figure 2:**
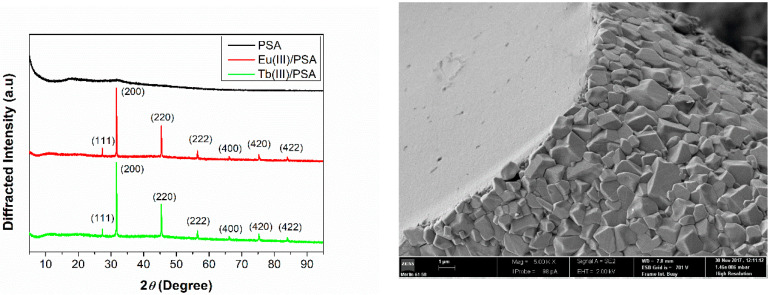
XRD patterns of PSA (black), Eu^3+^/PSA (red) and Tb^3+^/PSA (green), (**left**) and SEM micrograph of Eu^3+^/PSA freeze drying composite (**right**). Magnification 5000×.

A further insight into the interaction mechanism can also be gained from the analysis of lanthanoid/PSA solid composite. The XRD patterns of PSA, and Eu^3+^ and Tb^3+^ composites are shown in Figure 2. In the PSA diffraction pattern, we can see only a large shoulder diffuse peak, showing the amorphous structure of the polymer [36]. Unlike this, Eu^3+^/PSA and Tb^3+^/PSA diffractograms show a series of diffraction peaks at 2*θ* = 27.4, 31.5, 45.4, 56.5, 66.3, 75.4 and 83.9, and these peaks are assigned to the (111), (200), (220), (400), (420) and (422) plane of the NaCl-type structure [37]. This fact corroborates the occurrence of lanthanoid-PSA interaction, since the formation of NaCl is a consequence of the release and consequent interaction of counterions. It is also worth noting that no peak corresponding to the EuCl_3_ and TbCl_3_ crystal structure is observed, confirming that an interaction occurs, instead of a simple doping.

The SEM micrograph (Figure 2, right side) shows that the composite is covered with crystals, but the cross-section has a different and featureless morphology suggesting a different composition. Finally, the elemental mapping and EDX spectra (Figure S1) of Eu^3+^/PSA and Tb^3+^/PSA gel composites reveal a homogeneous distribution of lanthanoides through the polymer covered by a shell of NaCl.

### 3.2. Effect of Cations on the Luminescence Properties Lanthanoid/PSA Complexes

**Figure 3:**
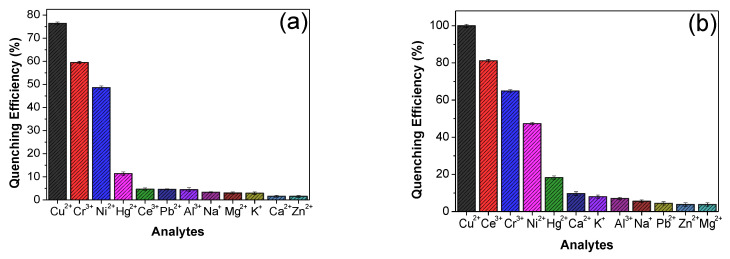
Quenching efficiency of (**a**) Eu^3+^/PSA (λ_em_ = 616 nm) and (**b**) Tb^3+^/PSA (λ_em_ = 545 nm) with different metal ions in aqueous solution.

As it was discussed in the previous section, Eu^3+^/PSA and Tb^3+^/PSA complexes have carboxyl groups available to coordinate metal ions, affecting their spectroscopic properties and making these composites potentially useful as luminescent probes for different ions. In consequence, the ability of these composites to detect different cations, in aqueous solution, has been tested by recording the luminescence emission spectra of Eu^3+^/PSA and Tb^3+^/PSA in the presence of different cations: Al^3+^, Ca^2+^, Ce^3+^, Cr^3+^, Cu^2+^, Hg^2+^, K^+^, Mg^2+^, Na^2+^, Ni^2+^, Pb^2+^ and Zn^2+^, as aqueous solutions of NO_3_^−^, Cl^−^ and SCN^−^ (Section 2.1). The results indicate that the luminescence intensity of the composites is strongly dependent on the metal ion species. Figure 3 shows the quenching efficiency of metal ions on the luminescence intensity of composite, defined as (*I*_0_ − *I*)/*I*_0_ × 100%, where *I*_0_ and *I* are the luminescence intensity without and with the addition of metal ions, respectively.

**Figure 4:**
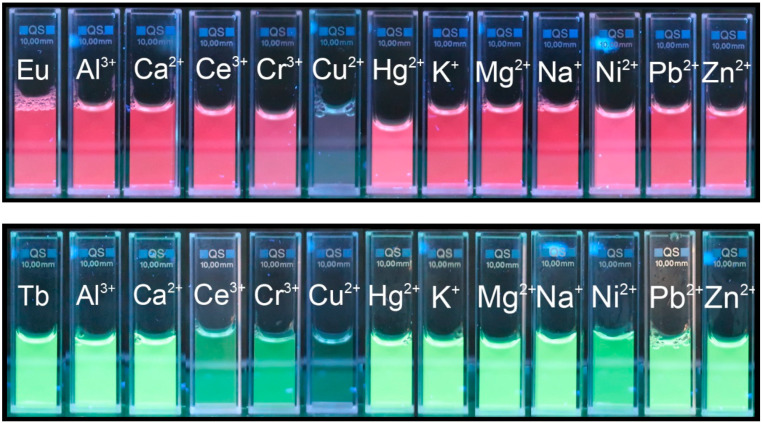
Fluorescence photographs of Eu^3+^/PSA (red) and Tb^3+^/PSA (green) alone (first to the left in each row), and in the presence of 3.33 mM of metal ions under UV light (365 nm) in aqueous solution.

Remarkably, Cu^2+^ is the most effective quencher for both composites. It completely quenches the emission of Tb^3+^/PSA at 545 nm (changing Tb^3+^/PSA emission from green to almost colorless in the presence of Cu^2+^ (Figure 4) and more than 70% of Eu^3+^/PSA emission at 616 nm. The emission of the latter one changed from red to light red, while the other metals did not have such a pronounced effect on luminescence intensity for Eu^3+^/PSA (Figure 4). This indicates that Tb^3+^/PSA possesses a higher sensitivity to Cu^2+^, being a promising sensor for the detection of Cu^2+^ ions. This behavior will be discussed in terms of the suppression mechanism. In general, the transitional metal ions display a stronger effect in luminescence compared to alkaline metal ions and alkaline earth metals. The effect arises from unpaired d-electrons found in transition metal ions contrasting to the closed shell electron configuration of group I and II metal cations [28,38].

### 3.3. Effect of Anions on Lanthanoid/PSA-Based Complex Properties

**Figure 5:**
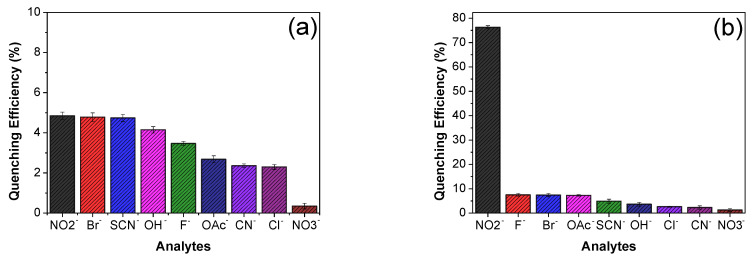
Quenching efficiency of (**a**) Eu^3+^/PSA (λ_em_ = 616 nm) and (**b**) Tb^3+^/PSA (λ_em_ = 545 nm) with different Na^+^ salts in aqueous solution.

In order to assess whether Eu^3+^/PSA and Tb^3+^/PSA composites can detect Cu^2+^ in the presence of other ions, the effects of several metal ions on the composite emission intensity were examined under the same experimental conditions. Firstly, the counter ion effect on the composite luminescence quenching was checked. For that, different sodium salts have been checked: Br^−^, Cl^−^, CN^−^, F^−^, NO_2_^−^, NO_3_^−^, OAc^−^, OH^−^ and SCN^−^. The results depicted in Figure 5 show no significant change in the luminescence emission intensity of Eu^3+^/PSA as the anion is varied (with variations around 5% at maximum, Figure 5 left) and, in consequence, it can be concluded that anions have no significant effect upon the detection of Cu^2+^ ions with Eu^3+^/PSA. 

In contrast, the luminescence emission of Tb^3+^/PSA composite is effectively quenched (75%) by nitrite ion. This can be attributed to energy transfer from Tb^3+^ to NO_2_^−^ [6] and, consequently NO_2_^−^ might be considered as an interferent for the Cu^2+^ detection for the Tb^3+^/PSA composite. The energy transfer may occur from Tb^3+^ to NO_2_^−^, due the ^5^*D*_4_ energy level of Tb^3+^ that matches with *T*_1_ energy level of NO_2_^−^; the same does not happen with Eu^3+^ [6].

In order to gain an insight into the potential selective detection of Eu^3+^/PSA and Tb^3+^/PSA composite toward Cu^2+^ in aqueous solution, the selectivity detection and the potential interference with other metal ions was studied by using Eu^3+^/PSA and Tb^3+^/PSA, and Cu^2+^ (3.33 M) in the presence of other metal ions (at equimolar concentrations). In both cases, the composite emission quenching by Cu^2+^ was hardly affected, as shown in Figure S2. The results indicate that Cu^2+^ detection is weakly perturbed by coexisting cations in solutions, confirming that the composites could selectively detect Cu^2+^, even in the presence of the other competing metal ions, clearly suggesting the potential of these composites as Cu^2+^ probes. The high selectivity of these composites towards Cu^2+^ could take place via interactions between functional sites, such as the uncoordinated carboxylic oxygen atom of PSA with Cu^2+^ ions, which will be discussed as follows.

### 3.4. On the Interaction Mechanism Between Cu(II) and Lanthanoid/PSA-Based Complexes

Having Cu^2+^ ions showed high interaction with composites, the nature of the quenching process should be unveiled. For that, the lifetimes of Eu^3+^/PSA and Tb^3+^/PSA composites were measured in absence, and in the presence of different concentrations of Cu^2+^. The decay curves for Eu^3+^/PSA and Tb^3+^/PSA in the presence of variable concentrations of Cu^2+^ were also monoexponencial, which indicates that Cu^2+^ does not replace the lanthanoids in the PSA chain, which would give rise to biexponential decays, due to the simultaneous emission of the composite and the free lanthanoids. 

The lifetimes fit properly the Stern-Volmer equation.

**Figure 6:**
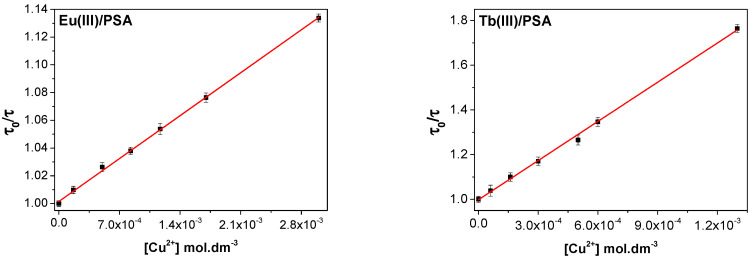
Stern-Volmer plot of Eu^3+^/PSA and Tb^3+^/PSA lifetimes with different Cu^2+^ concentrations.

*τ*_0_*/τ* = 1 + *K*_SV,D_[Q] (1)

where *τ*_0_ and *τ* are the composite lifetime before and after the incorporation of the metal cation respectively, *K*_SV,D_ is the dynamic Stern-Volmer constant and [Q] = [Cu^2+^], as shown in Figure 6. The computed dynamic Stern-Volmer constants are equal to 44.0 (±0.5) and 583 (±7) M^−1^ for Eu^3+^/PSA and Tb^3+^/PSA, respectively, which are an order of magnitude lower than the ones obtained with the emission intensities Stern-Volmer plot (580 and 5655 M^−1^, for the same composites, respectively). These results confirm that both static and dynamic quenching are simultaneously taking place [11,26,39,40].

The Cu^2+^ paramagnetism contributes to the quenching of the fluorescence emission by increasing the intersystem crossing process [29,41,42,43,44]. The higher Tb^3+^/PSA sensitivity to Cu^2+^ with respect to that of Eu^3+^/PSA could be partially due to the overlap between the absorption spectra of both Tb^3+^/PSA and Cu^2+^ (spectra not shown), which is likely to significantly diminish Tb^3+^/PSA emission intensity [28,45,46]. In general, PSA plays an important role in the quenching process, due to its ability to coordinate Cu^2+^ ions. This interaction can enhance the proximity between the composites and Cu^2+^ promoting a dynamic quenching and, simultaneously, form a new non-luminescent composite. We hypothesize that the polymer may act as a surface, which promotes sensor luminescence quenching by adsorbing Cu^2+^ ions through the complexation with the polymer carboxylic groups, as will be confirmed by EDX mapping [47]. As stated before, such an interaction can also be used for the application of these composites on the selective removal of Cu^2+^ as an added value metal.

### 3.5. Effect of pH and Quenching Rate

The emission intensity of Eu^3+^/PSA/Cu^2+^ and Tb^3+^/PSA/Cu^2+^ composites, with three different Cu^2+^ concentrations for each system, were recorded at 616 and 545 nm at several delays after sample preparations (Figure S3), showing that, independently of Cu^2+^ concentrations, the emission intensity reaches an equilibrium almost instantaneously (around a minute), remaining constant for almost a week [48], showing the great stability of the composites. Additionally, the emission intensities of Eu^3+^/PSA/Cu^2+^ and Tb^3+^/PSA/Cu^2+^ composites, at 616 and 545 nm, respectively, recorded for solutions with different pH values (Figure S4) were approximately the same, proving that composites were stable over a wide pH range (6–11), and indicating their potential applications on a large pH range, including environmental and physiological conditions.

### 3.6. Sensitivity of Eu^3+^/PSA and Tb^3+^/PSA to Cu^2+^

**Figure 7:**
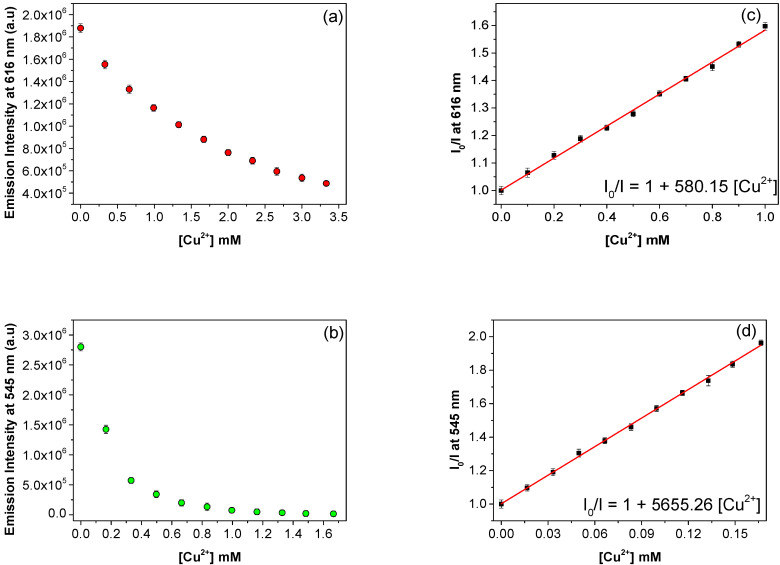
Emission intensities of (**a**) Eu^3+^/PSA (^5^*D*_0_ → ^7^*F*_2_) transition, (**b**) Tb^3+^/PSA (^5^*D*_4_ → ^7^*F*_5_) transition and Stern-Volmer plots of (**c**) Eu^3+^/PSA and (**d**) Tb^3+^/PSA with lower concentrations of Cu^2+^.

Sensitivity is a key factor to evaluate the performance of a probe to determine traces of an analyte and also for remediation purposes. To further examine the Eu^3+^/PSA and Tb^3+^/PSA composite sensing sensitivity towards Cu^2+^, their luminescence emission quenching by CuCl_2_ (at concentrations ranging from 0.33 mM to 3.33 mM) were studied. The emission luminescence spectra of composites are shown in Supplementary Information (Figures S5–S8). Moreover, Figure 7a shows the emission luminescence intensity of Eu^3+^/PSA, at 616 nm which gradually decreases with Cu^2+^ concentration. When the concentration 3.33 mM Cu^2+^ is reached, the quenching efficiency is above 75%.

Figure 7c shows the ratio of emission intensities in absence (*I*_0_) and presence of Cu^2+^ (*I*) of ^5^*D*_0_–^7^*F*_2_ emission at 616 nm versus the concentration of Cu^2+^ for metal ion concentrations lower than 10^−3^ M. In this concentration range, Eu^3+^/PSA emission quenching follows the Stern-Volmer equation (Equation (1)). A good determination coefficient (*R*^2^ = 0.997) was obtained for the fitting of Equation (1) to experimental data, leading to a Stern-Volmer constant, *K*_SV_, equal to 580 (±1) M^−1^. The limit of detection (LOD) for ions Cu^2+^ has been calculated by using the equation: 3*σ*/*k*, where *σ* is the standard error and *k* the slope. The LOD has been calculated as 3.06 × 10^−5^ M (corresponding to 1.94 ppm). Therefore, Eu^3+^/PSA presents an excellent sensitivity towards Cu^2+^. Similar sensitivity tests were carried out with Tb^3+^/PSA composites, and results are shown in Figure 7b,d. The fluorescence intensity at 545 nm is drastically quenched by the gradual increase of the concentration of Cu^2+^ and for Cu^2+^ concentrations below 1.8 × 10^−3^ M, the Stern-Volmer equation is followed (Figure 7d). The calculated *K*_SV_ value is 5655 (±68) M^−1^ and the limit of detection for Cu^2+^ is 3.56 × 10^−^^6^ M (0.226 ppm), indicating a higher sensitivity to Cu^2+^ when compared to Eu^3+^/PSA. K_SV_ values obtained for Cu^2+^ in both composites are relatively high, and can be compared with the values obtained for other compounds previously used for Cu^2+^ detection (Table 2). This indicates that both composites have great potential for application in Cu^2+^ ion sensing, especially Tb^3+^/PSA, which shows greater sensitivity.

### 3.7. Characterization of Solid Lanthanoid/PSA Composites Containing Cu^2+^

**Figure 8:**
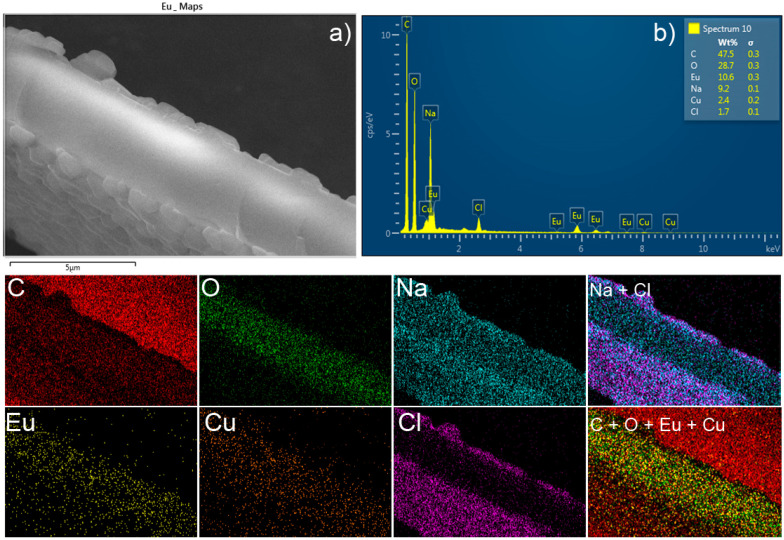
(**a**) SEM micrography, (**b**) EDX spectra and elemental maps of Eu^3+^/PSA after contact with Cu^2+^. The right-hand figures represent the combined elemental maps; for the sake of clarity, the elemental maps of counterions (Na and Cl) are represented in a separated figure.

SEM micrographs of the composites obtained with the objective of investigating the morphology and structure of the composites after contact with Cu^2+^ (Figure S9), indicate that the structure maintains its integrity and, therefore, the quenching effect is not a consequence of structure collapse. The EDX spectra of composite, obtained after immersion in a Cu^2+^ solution, show the presence of these ions, suggesting a strong interaction between Cu^2+^ and the composites. To support this hypotheses, elemental EDX maps have been made, and confirm the interaction of Cu^2+^ with the composites in the region where the composites are not coated with NaCl crystals. This also shows that most of the Cu^2+^ added is bound to the composites having a uniform distribution in the same region that Eu and Tb (Figure 8 and Figure S10, respectively). From the elemental maps, we can also conclude that in the Eu^3+^/PSA and Tb^3+^/PSA composites a simultaneous interaction of the polymer with both the lanthanoid and Cu^2+^ ions is produced; i.e., Cu^2+^ ions do not replace the lanthanoides in the PSA chain, in good agreement with the previously discussed lanthanoids lifetime results. The surface of the composites is characterized by a high content of oxygen, making available sites of interaction with Cu^2+^ and other metallic ions. We can therefore suggest that the main mechanism of the composite luminescent quenching induced by metal ions is the Cu^2+^ complexation, with the composite through the carboxylate groups of Ln^3+^/PSA. 

## 4. Conclusions

Two highly luminescent water soluble stable metal-organic composites (Eu^3+^/PSA and Tb^3+^/PSA) were prepared, showing a significant emission quenching in the presence of Cu^2+^, when compared with a set of other metal ions: Al^3+^, Ca^2+^, Ce^3+^, Cr^3+^, Hg^2+^, K^+^, Mg^2+^, Na^+^, Ni^2+^, Pb^2+^ and Zn^2+^ that, in general, do not compete with copper ions for the composite. Polymer composite emission intensities and lifetimes in the presence of Cu^2+^ follow Stern-Volmer kinetics, indicating that both static and dynamic quenching processes take place simultaneously, with the former being one order of magnitude higher for both composites. This probes that complex formation is the main interaction mechanism between the lanthanoid/PSA composites and Cu^2+^, likely through the polymer carboxylate groups. The composite shows a fast response to the presence of Cu^2+^ (less than one minute) and linear for Cu^2+^ concentration below 1 mM (Eu^3+^/PSA) and 1.8 × 10^−3^ M (Tb^3+^/PSA), within an extended pH range (6 to 11), and with detection limits of 1.94 and 0.22 ppm, in the case of Eu^3+^/PSA and Tb^3+^/PSA, respectively. In our experimental conditions, no competition has been detected between the lanthanides and copper ions, indicating that the PSA has carboxylate groups available for coordination after the interaction with lanthanoid ions. Additionally, nitrite ions also promote high quenching efficiency for Tb^3+^/PSA composite through an energy transfer process. The experimental results reported have unraveled a new promising method for a simple and reliable monitoring or removal of Cu^2+^ from different media. Moreover, these composites can be used both as aqueous solution and in a gel state, enlarging the potential range of their practical applications.

## Figures and Tables

**Table 1:** Lifetimes (*τ*), decay constant (*k*) and number of coordinated water molecules (*n*) of Eu^3+^ and Tb^3+^ in water and D_2_O and in poly(sodium acrylate) (PSA) aqueous and D_2_O solutions.

Sample	Solvent	*τ* (μs)	*k* (ms^−1^)	*n* (H_2_O Coordinated)
EuCl_3_	H_2_O	112	8.92	9
EuCl_3_	D_2_O	1775	0.56	
Eu^3+^/PSA	H_2_O	274	3.65	3
Eu^3+^/PSA	D_2_O	1602	0.62	
TbCl_3_	H_2_O	431	2.32	9
TbCl_3_	D_2_O	3565	0.28	
Tb^3+^/PSA	H_2_O	861	1.16	3
Tb^3+^/PSA	D_2_O	3002	0.33	

**Table 2:** Stern-Volmer constant for the quenching of the emission of several lanthanoid-based sensors by Cu^2+^.

Luminescent Material	*K*_sv_ (M^−1^)	Reference
[Eu(pdc)_1.5_(dmf)]·(DMF)_0.5_(H_2_O)_0.5_	89	[49]
Eu^3+^/PSA	580	This Work
{[Eu_2_(abtc)_1.5_(H_2_O)_3_(DMA)]·H_2_O·DMA}_n_	529	[50]
{[Eu(HL)(L)(H_2_O)_2_]_2H_2_O}n	116	[43]
{[Eu(L)(ox)_0.5_(H_2_O)_2_]_H_2_O}n	2074	[51]
Tb^3+^/PSA	5655	This work
Tb-SA	6298	[52]

## Supplementary Materials

The following are available online at https://www.mdpi.com/2073-4360/12/6/1314/s1, Figure S1: EDX spectra and elemental maps of freeze-dried Eu^3+^/PSA (left) and Tb^3+^/PSA (right), Figure S2: Comparison of emission intensity of Eu^3+^/PSA at 616 nm (left) and Tb^3+^/PSA at 545 nm (right) of composites alone (*I*_0_) and interacting with different metal ions in aqueous solution under the same conditions (*I*), Figure S3: Emission intensities of (A) Eu^3+^/PSA at 616 nm (left) and (B) Tb^3+^/PSA at 545 nm (right) with different concentration of Cu^2+^ ions in aqueous solution at several delays after sample preparation, Figure S4: Effects of pH on the emission intensities of (A) Eu^3+^/PSA at 616 nm and (left) (B) Tb^3+^/PSA at 545 nm (right), without Cu^2+^ (black) and with 3.33 mM of Cu^2+^ (red), Figure S5: Emission spectra of Eu^3+^/PSA, ^5^*D*_0_ → ^7^*F*_2_ transition emission intensity at 616 nm (inset) of Eu^3+^/PSA with different concentrations of Cu^2+^, T S6: Emission spectra of Eu^3+^/PSA and Stern-Volmer plot of ^5^*D*_0_ → ^7^*F*_2_ transition at 616 nm (inset) for Eu^3+^/PSA with lower concentrations of Cu^2+^, Figure S7: Emission spectra of Tb^3+^/PSA and ^5^*D*_4_ → ^7^*F*_5_ transition intensities at 545 nm (inset) for Tb^3+^/PSA with different concentrations of Cu^2+^, Figure S8: Emission spectra of Tb^3+^/PSA and Stern-Volmer plot of ^5^*D*_4_ → ^7^*F*_5_ transition at 545 nm (inset) for Tb^3+^/PSA with lower concentrations of Cu^2+^, SEM micrographs of (a) Eu^3+^/PSA, (b)Tb^3+^/PSA before contact with Cu^2+^ and (c) Eu^3+^/PSA, (d) Tb^3+^/PSA after contact with Cu^2+^. Magnification ×500, Figure S10: (a) SEM micrography, (b) EDX spectra and elemental maps of Tb^3+^/PSA after contact with Cu^2+^.
