# Supplementary material for: Luminescent Properties of Lanthanoid-Poly(Sodium Acrylate) Composites: Insights on the Interaction Mechanism
Source: Polymers (Basel). 2020 Jun 9;12(6):1314. doi: 10.3390/polym12061314 (PMC7362023; doi:10.3390/polym12061314)
Supplement: Supplementary file 1 [file polymers-12-01314-s001.pdf]

polymers

MDPI

Article

Supporting Information

Luminescent Properties of Lanthanoid-Poly(Sodium Acrylate) Composites: Insights on the Interaction Mechanism

Alan F. Y. Matsushita [1], María José Tapia [2], Alberto A. C. C. Pais [1] and Artur J. M. Valente [1,*]

[1] CQC, Department of Chemistry, University of Coimbra, 3004-535 Coimbra, Portugal; alanmatsushita@hotmail.com (A.F.Y.M.); pais@ci.uc.pt (A.A.C.C.P.)

[2] Department of Chemistry, Universidad de Burgos, 09001 Burgos, Spain; mjtapia@ubu.es

* Correspondence: avalente@ci.uc.pt; Tel.: +351-239-852-080

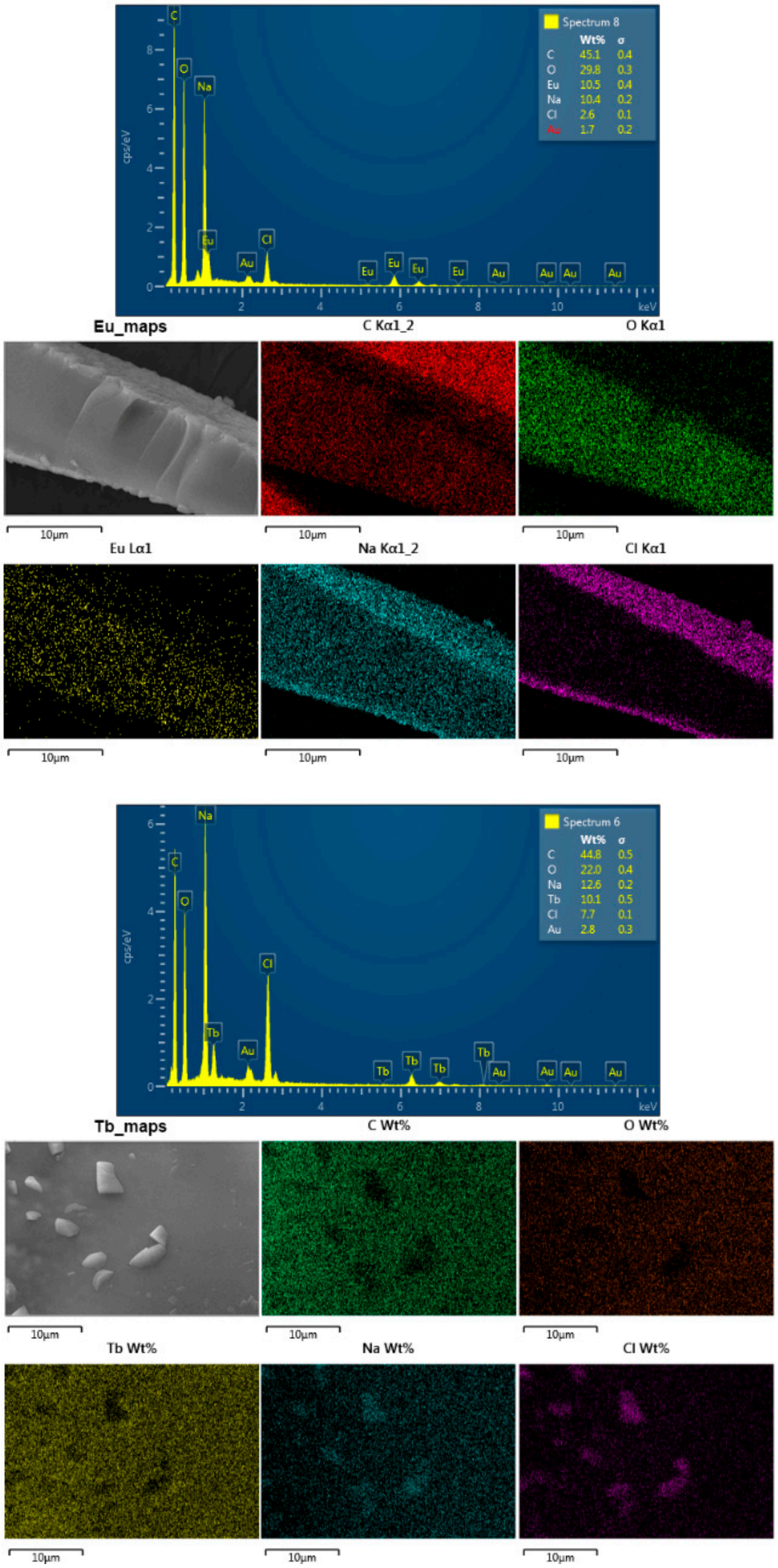

Figure S.1. EDX spectra and elemental maps of freeze-dried Eu^{3+}/PSA (top) and Tb^{3+}/PSA (bottom).

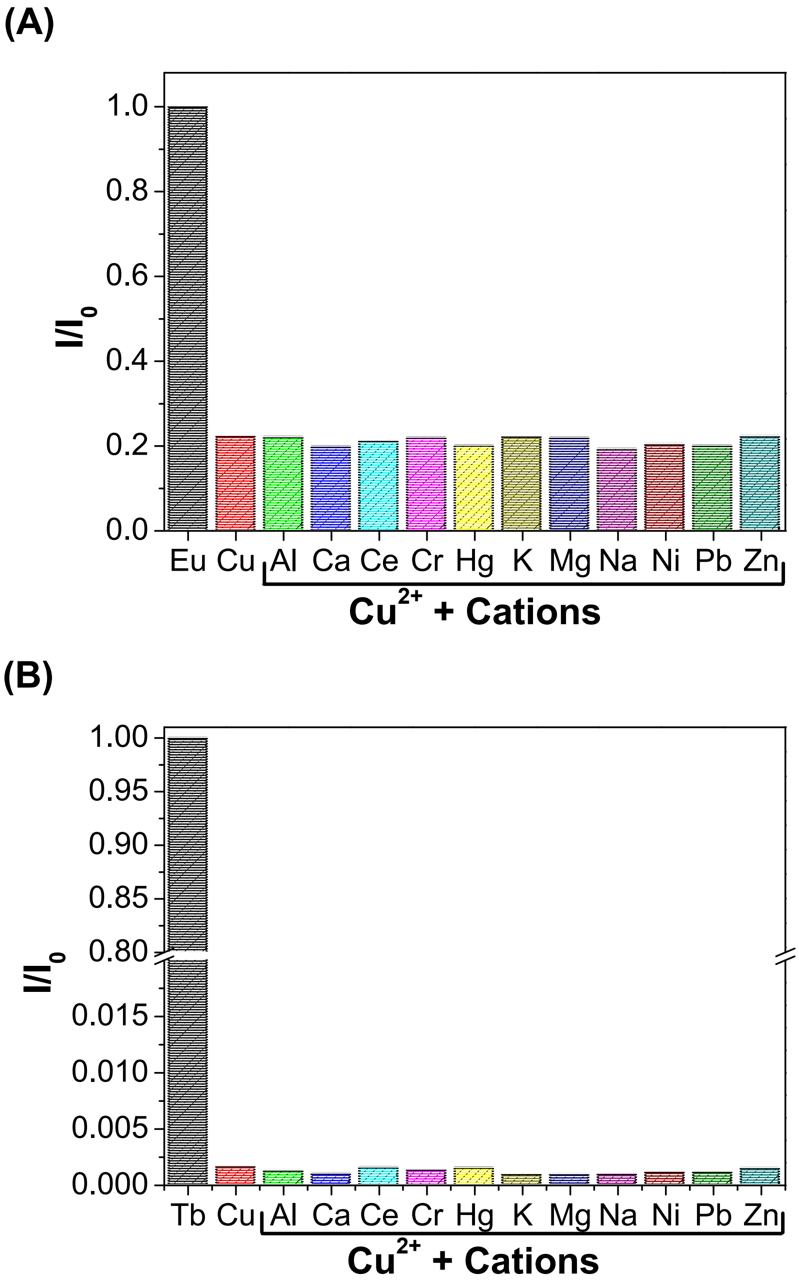

Figure S.2. Comparison of emission intensity of Eu^{3+}/PSA at 616 nm (A) and Tb^{3+}/PSA at 545 nm (B) of composites alone (I_0) and interacting with different metal ions in aqueous solution under the same conditions (I).

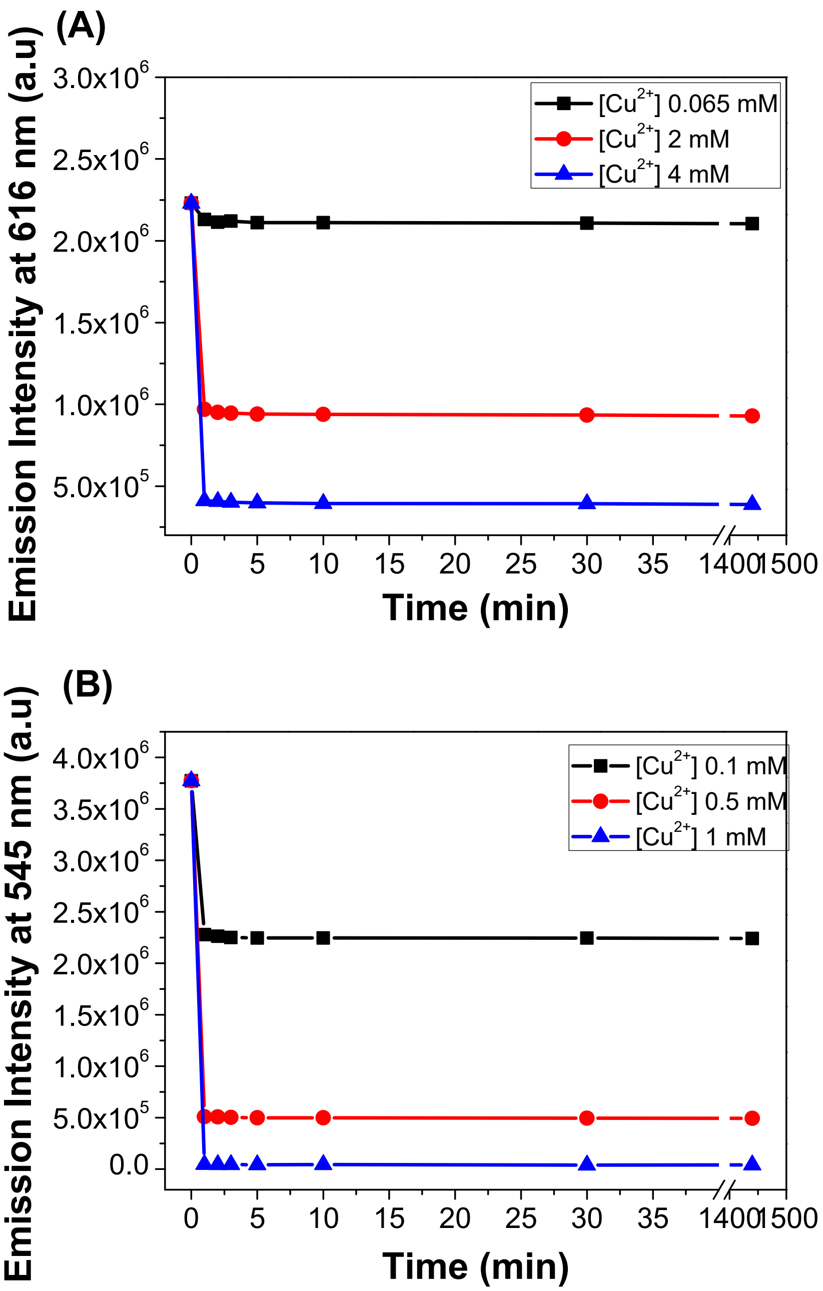

Figure S.3. Emission intensities of (A) Eu^{3+}/PSA at 616 nm and (B) Tb^{3+}/PSA at 545 nm with different concentration of Cu^{2+} ions in aqueous solution at several delays after sample preparation.

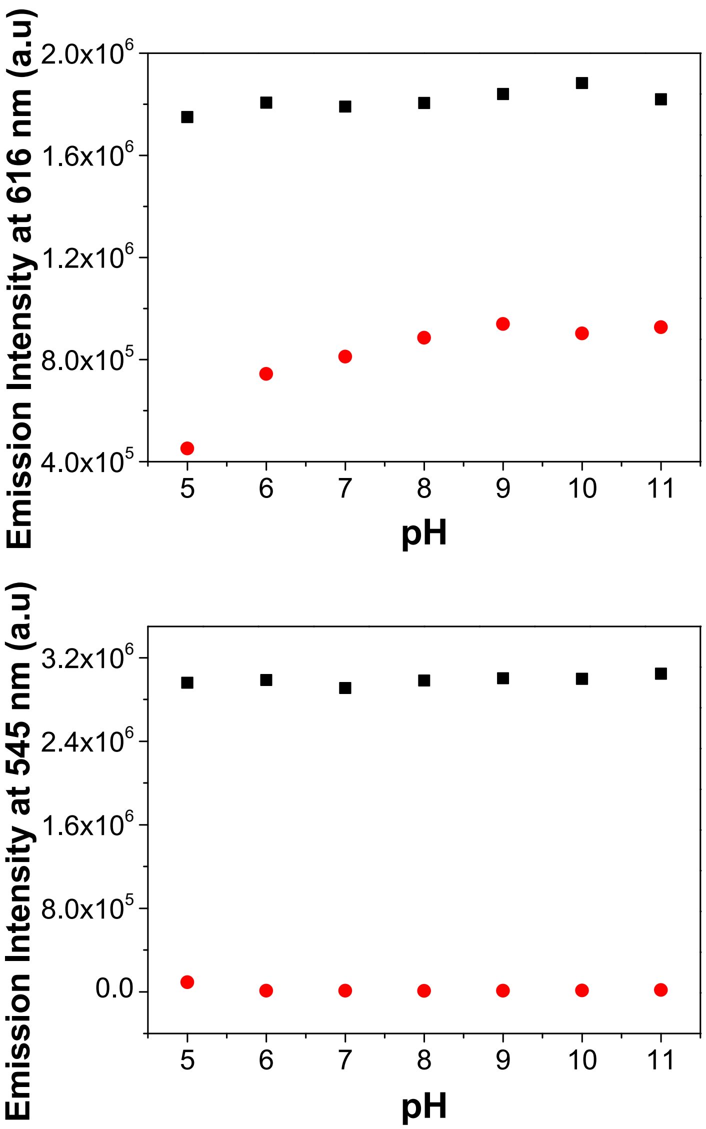

Figure S.4. Effects of pH on the emission intensities of Eu^{3+}/PSA at 616 nm (top) and Tb^{3+}/PSA at 545 nm (bottom), without Cu^{2+} (black) and with 3.33 mM of Cu^{2+} (red).

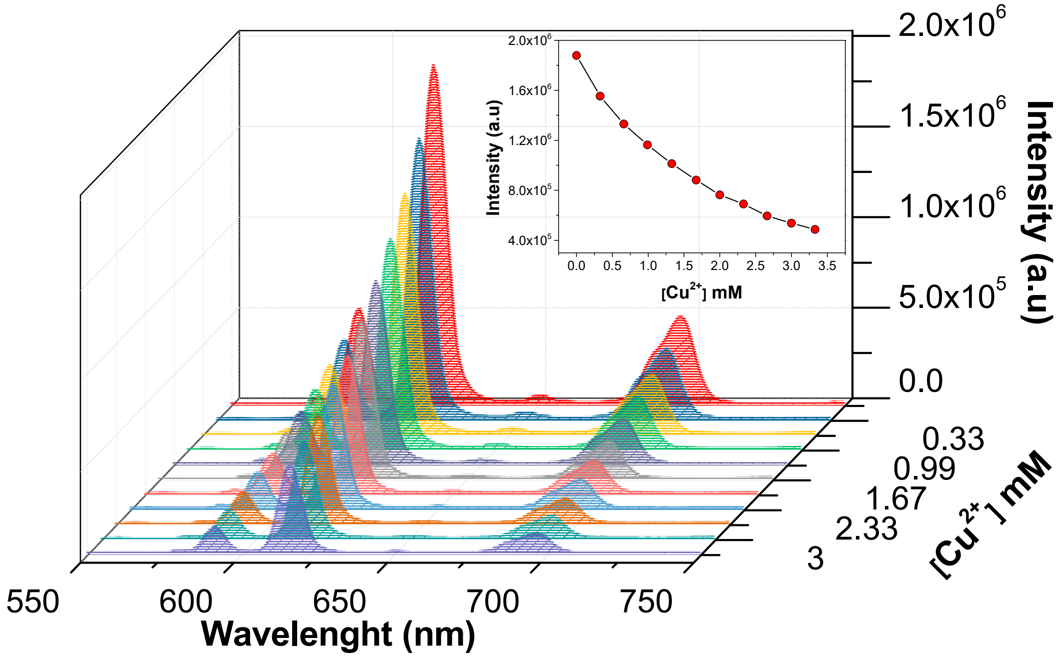

Figure S.5. Emission spectra of Eu^{3+}/PSA, $^{5}D_{0} \rightarrow {}^{7}F_{2}$ transition emission intensity at 616 nm (inset) of Eu^{3+}/PSA with different concentrations of Cu^{2+}.

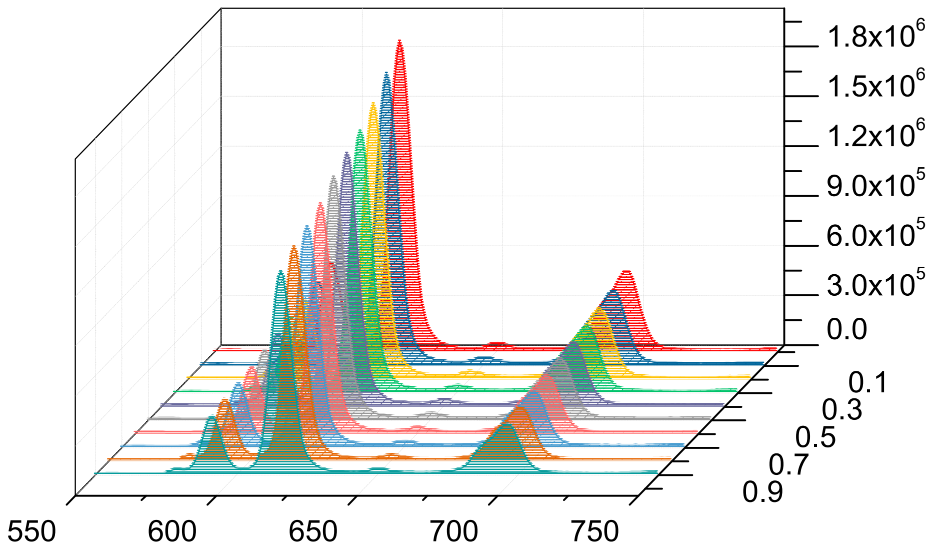
1.8x10^6
1.5x10^6
1.2x10^6
9.0x10^5
6.0x10^5
3.0x10^5
0.0
0.1
0.3
0.5
0.7
0.9
550
600
650
700
750
